# The pharmacokinetic and pharmacodynamic properties and short-term outcome of a novel once-weekly PEGylated recombinant human growth hormone for children with growth hormone deficiency

**DOI:** 10.3389/fendo.2022.922304

**Published:** 2022-08-11

**Authors:** Yan Liang, Cai Zhang, Haiyan Wei, Hongwei Du, Gaixiu Zhang, Yu Yang, Hua Zhang, Haihong Gong, Pin Li, Fuying Song, Zhuangjian Xu, Ruoyi He, Weidong Zhou, Heng Zheng, Li Sun, Xiaoping Luo

**Affiliations:** ^1^ Department of Pediatrics, Tongji Hospital, Tongji Medical College, Huazhong University of Science and Technology, Wuhan, China; ^2^ Department of Endocrinology, Genetics and Metabolism, Children’s Hospital Affiliated to Zhengzhou University, Henan Children’s Hospital, Zhengzhou Children’s Hospital, Zhengzhou, China; ^3^ Department of Pediatrics, The First Affiliated Hospital, Jilin University, Changchun, China; ^4^ Department of Pediatric Endocrinology, Shanxi Provincial Children’s Hospital, Taiyuan, China; ^5^ Department of Endocrinology, Genetics and Metabolism, Jiangxi Provincial Children’s Hospital, Nanchang, China; ^6^ Department of Pediatrics, Sanya Central Hospital, Sanya, China; ^7^ Department of Pediatrics, The First Affiliated Hospital of Nanjing Medical University, Nanjing, China; ^8^ Department of Endocrinology, Children’s Hospital of Shanghai, Shanghai Jiao Tong University, Shanghai, China; ^9^ Department of Endocrinology, Children’s Hospital, Capital Institute of Pediatrics, Beijing, China; ^10^ Department of Pediatrics, Wuxi Fourth People’s Hospital, Wuxi, China; ^11^ Office of General Manager, Xiamen Amoytop Biotech Co., Ltd, Xiamen, China; ^12^ Department of Pharmacy, Tongji Hospital, Tongji Medical College, Huazhong University of Science and Technology, Wuhan, China

**Keywords:** long-acting growth hormone, Y-shape branched PEGylation, growth hormone deficiency, prepubertal children, clinical trial

## Abstract

**Objectives:**

To investigate the pharmacokinetics (PK) and pharmacodynamics (PD) of Y-shape branched PEGylated recombinant human growth hormone (YPEG-rhGH) and evaluate its short-term efficacy and safety in children with growth hormone deficiency (GHD).

**Methods:**

A total of 43 children with GHD from 12 sites in China were enrolled in this randomized, multicenter, active-controlled, double-blind (YPEG-rhGH doses) trial. Patients were randomized 1:1:1:1 to 100, 120, and 140 μg/kg/week of YPEG-rhGH groups and daily rhGH 35 μg/kg/day groups. The treatment lasted 12 weeks. The primary outcome was the area under the curve of the change of insulin-like growth factor-1 (IGF-1). The secondary outcome was the height velocity (HV) increment at week 12.

**Results:**

A dose-dependent response of maximum plasma concentration (C_max_) and area under the concentration-time curves from 0 to 168 hours (AUC_0-168h_) were observed for YPEG-rhGH. The ratio of C_max_ and the ratio of AUC_0-168h_ from the first to the last dosing were 1.09~1.11 and 1.22~1.26 respectively. A YPEG-rhGH dose-dependent increase in area under effect curve (AUEC) of IGF-1 fold change was observed. Model-derived mean IGF-1 SDS was in the normal range for all three YPEG-rhGH doses. At week 12, HV was 7.07, 10.39, 12.27 cm/year, and 11.58 cm/year for YPEG-rhGH 100, 120, and 140 μg/kg/week and daily rhGH respectively. Adherence and safety were consistent with the profile of daily rhGH. No related serious adverse events were reported.

**Conclusion:**

The PK/PD suggests that YPEG-rhGH is suitable for the once-weekly treatment of pediatric GHD. YPEG-rhGH 120 ~ 140 μg/kg/week provides the closest HV increment with similar safety and tolerability compared to daily rhGH 35 μg/kg/day in children with GHD.

**Clinical Trial Registration:**

ClinicalTrials.gov, identifier [NCT04513171].

## Introduction

Recombinant human growth hormone (rhGH) has been used to treat children with growth hormone deficiency (GHD) since 1985 (1). The long-term safety and efficacy of rhGH in children with GHD and other diseases have been well investigated (2, 3). Studies have suggested that various factors could affect the therapeutic effect of rhGH, including the patient’s age, the dosage of rhGH, and the treatment adherence (4–6). In addition, pain at injection sites or needle phobia and the inconvenience of daily injections may lead to frequent dose omissions or treatment cessation, ultimately affecting the therapy outcomes (7, 8). Thus, it has been postulated that long-acting growth hormone (LAGH) with a lower injection frequency could improve adherence and treatment outcomes.

LAGH preparations include depot formulations, PEGylated formulations, pro-drug formulations, non-covalent albumin binding GH, and GH fusion proteins (9). For the treatment of pediatric GHD, only three LAGH analogs have been approved: lonapegsomatropin-tcgd (Skytrofa^®^), a long-acting prodrug of hGH produced by recombinant DNA technology, has been approved in the United States, while Eutropin Plus^®^ (a depot formulation) and Jintrolong^®^ (PEGylation of rhGH), were approved for the Asian market (10–12). PEGylation is a process that conjugates polyethylene glycol (PEG) moieties with a protein, which increases protein stability, and reduces antigenicity and renal clearance (13). In order to reduce immunogenicity and increase the half-life of drugs, branched PEGylated formulations are often used, such as U-shape and Y-shape PEGylation (14, 15). Experimental conditions, including pH, temperature and reaction time are critical for the PEGylation process, especially for the selective modification of specific amino acids in the biomolecules (16).

Jintrolong^®^ is a U-shape branched N-terminal PEGylated rhGH and the only once-weekly PEGylated rhGH approved for pediatric GHD (11). It was developed by a reaction system with a pH of 6.5-7.0, resulting in the modification of the α-amino group in the N-terminal phenylalanine of rhGH (17). More recently, a novel Y-shape branched PEGylated rhGH (YPEG-rhGH) was formulated with the conjugation of a 40 kDa Y-shape branched PEG chains to the α-amino group in the ϵ-amino group in the side chain of lysine of rhGH in a reaction system with the pH of 10.5 (16). Our previous study showed that the cellular bioactivity of YPEG-rhGH was twice of that of N-terminal modification, and the half-life was 20 times longer than that of daily rhGH (18). Thus, we concluded that YPEG-rhGH might be potentially used as a long-acting growth hormone for pediatric GHD patients.

A recent phase 1 trial in 36 healthy male adults showed the potential once-weekly administration of YPEG-rhGH, indicated by the mean half-life of 65-120 hours and consistent tolerability and safety with daily rhGH (data no shown). In this study, we further investigated the pharmacokinetic/pharmacodynamic (PK/PD) profiles and the short-term clinical outcomes of YPEG-rhGH in children with GHD.

## Materials and methods

### Study design

The aim of this study was to develop a pharmacokinetic/pharmacodynamic model to predict YPEG-rhGH exposure and insulin-like growth factor-I (IGF-I) response and investigate the short-term clinical outcomes in children with GHD. This randomized, multicenter, active-controlled and double-blind (YPEG-rhGH doses) trial was conducted at 12 sites in China between March 2019 and January 2020. The protocol was approved by the Ethics Committee of Tongji Hospital, Tongji Medical College, Huazhong University of Science and Technology, and the study was conducted in accordance with the Declaration of Helsinki. Informed consent was obtained in writing from the parents/guardians, and child assent was obtained as age-appropriate.

### Patients

A total of 40 patients with idiopathic GHD were planned for enrollment. The inclusion criteria were as follows (1): confirmed diagnosis of GHD before the screening, defined as height standard deviation score (HTSDS) <−2 based on the Chinese general population standard for age (19), height velocity (HV) ≤5.0 cm/year, bone age (BA) delayed more than 2 years compared to the chronological age (CA), GH peak level <10 ng/mL provoked by two different medications, and insulin-like growth factor-1 (IGF-1) level lower than the age-appropriate medium level (2); older than 3 years of age; prepubertal status (Tanner stage 1 for pubic hair and testis volume < 4 mL in boys, Tanner stage 1 for breast development and pubic hair in girls), 10 years or younger for girls, and 11 years or younger for boys (3); proportionate short stature with normal intelligence.

Exclusion criteria were (1): previous administration of rhGH and sex hormone treatments (2); prior history or presence of malignancy and/or intracranial tumor (3); severe allergic constitution (4); other patterns of growth abnormalities, such as idiopathic short stature, Turner Syndrome, thyroid hormone deficiency, hypoadrenalism and antidiuretic hormone deficiency (5); other clinical conditions that may impair growth such as liver dysfunction, diabetes, malnutrition, and deformities (6); Cobb’s angle > 15 degrees in the patients with scoliosis.

### Study protocol

Eligible subjects were randomly assigned to receive one of three subcutaneous doses of YPEG-rhGH (2 mg/0.5 mL, Xiamen Amoytop Biotech Co., Ltd.), *i.e.*, 100, 120, and 140 μg/kg/week, or daily rhGH (Norditropin, Novo Nordisk) at a dose of 35 μg/kg/day. All patients were assessed at baseline and 4 and 12 weeks after the first dosing. At each assessment, height and weight were monitored, and adverse events (AEs) were reported. The AEs were classified according to Common Terminology Criteria for Adverse Events (CTCAE) Version 5.0 published by National Cancer Institute (NCI). The treatment adherence was evaluated by ratio of the number of reported dosing to planned dosing multiplied by 100.

Blood samples were collected at baseline and 4 and 12 weeks for the PK/PD profile of YPEG-rhGH. IGF-1 was determined by ELISA kits (Siemens Catalog # L2KGF2, RRID: AB_2756880). IGF-I standard deviation score (SDS) was calculated according to previously reported modified least mean squares model (20). Free thyroxine (T4), thyroid-stimulating hormone (TSH), calcium, phosphate, lipids, glycated hemoglobin (HbAlc), insulin, fasting blood glucose, complete blood count, liver and renal function were tested at baseline and after 12 weeks of the treatments. The primary outcome was the area under the curve of the change of insulin-like growth factor-1 (IGF-1). The secondary outcome was the height velocity (HV) increment at week 12.

### Random assignment

Prepubertal patients with GHD were randomly assigned in a 1:1:1:1 ratio to 100, 120, and 140 μg/kg/week YPEG-rhGH and daily rhGH 35 μg/kg/day. The randomization was conducted by an interactive web response system, and stratified by sex and peak GH level (≤5 ng/mL or > 5 ng/mL). The trial was double-blind with regard to YPEG-rhGH dose, and the clinical assessments were conducted by assessors who were blinded to YPEG-rhGH dosing.

### Population pharmacokinetic/pharmacodynamic modeling

The serum samples used to assess the pharmacokinetic/pharmacodynamic (PK/PD) profile of YPEG-rhGH were collected 30 minutes before dosing, 11 ± 3 hours and 96 ± 3 hours after dosing, and 30 minutes before next dosing at the first and 12th injection, as well as 11 ± 3 hours after the 4th injection. IGF-1 levels and YPEG-rhGH concentrations were measured for each sample to construct the PK/PD model. To adjust the differences in basal IGF-1 levels between groups, fold change of measured IGF-1 levels with respect to the baseline IGF-1 level (IGF-1 FC) was chosen as the PD parameter

### Statistical analysis

Population PK/PD modeling was performed in NONMEM version 7.2 (ICON Development Solutions), PsN version 4.2.0, and R version 3.4.0. The patients who were randomly assigned and received at least one dose of treatment were included in the full analysis set (FAS). Missing data were imputed by the last-observation-carried-forward (LOCF) method. The safety analysis set (SAS) contained all randomly assigned children who received at least one treatment.

SAS 8.1(SAS Institute, Inc., Cary, NC, USA) was used for statistical analyses. For normally distributed data, unpaired 2-tailed Student’s t-test and one-way analysis of variance were used. For data with non-normal distribution, the Wilcoxon Rank Sum Test was used for 2-group and multiple group comparisons, respectively. Enumerated data were compared using the Chi-square test and the Fisher’s exact test. Data were expressed as mean ± s.d. with significance set at *P* < 0.05.

## Results

### Population pharmacokinetic/pharmacodynamic model derived profile of YPEG-rhGH

A total of 751 PK samples and 818 IGF-I samples from 67 subjects treated with YPEG-rhGH (540 samples from 36 subjects in the phase 1 trial and 287 samples from 31 patients in this study) were included in the analysis, after excluding 76 PK data that were below the limit of quantification and 8 IGF-1 samples from one subject whose basal IGF-1 level was below the limit of quantification.

#### Model-derived pharmacokinetic profile of YPEG-rhGH

**Figure 1:**
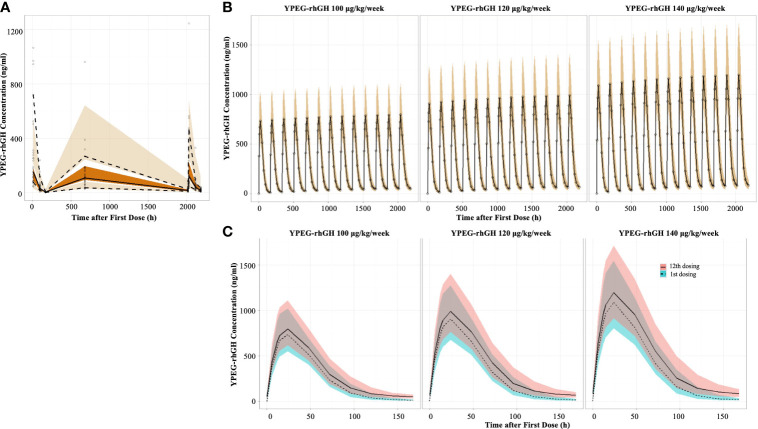
Model-derived PK profiles of YPEG-rhGH **(A)**, Prediction-corrected visual predictive checks (pcVPC) of the YPEG-rhGH concentration. The hollow dots indicated the prediction-corrected YPEG-rhGH concentration. The top dashed line, the middle solid line and the bottom dashed line indicated 10th percentile, median and 90th percentile of prediction-corrected YPEG-rhGH concentrations. The top, middle and bottom area indicated the 95% confidence interval of each line. **(B)**, model-derived PK profiles of YPEG-rhGH in the 12-week treatment period. The hollow dots and solid line indicated the median of YPEG-rhGH concentrations. The shallow area indicated the 5% and 95% confidence intervals predicted by the model. **(C)**, model-derived PK profiles of YPEG-rhGH within one treatment interval after the first and last dosing. The green and pink area indicated the 95% confident interval of YPEG-rhGH concentration after the first and last dosing.

Prediction-corrected visual predictive checks (pcVPC) demonstrated good prediction of YPEG-rhGH concentration (**Figure 1A**). The PK profile of YPEG-rhGH slightly increased after multiple dosing, and reached a steady state at week 12 in YPEG-rhGH 100, 120 and 140 μg/kg/week groups (**Figure 1B**). As described in **Table 1**, the median time to reach maximum concentration (T_max_) was 24 h for YPEG-rhGH 100, 120 and 140 μg/kg/week after the first dosing, which was the same as the median T_max_ of the last dosing, suggesting that multiple dosing does not change the absorption of YPEG-rhGH in GHD children.

**Table 1:** The model derived PK parameters of YPEG-rhGH.

Dose (μg/kg/week)	Week 1				Week 12				RC_max_	RAUC_0-168h_
	N	C_max_ (ng/ml)	AUC_0-168h_ (ng·h/ml)	T_max_ (h)	N	C_max_ (ng/ml)	AUC_0-168h_ (ng·h/ml)	T_max_ (h)		
100	30	746 (20.9)	43500 (19.9)	24 (24,24)	30	811 (20.6)	53200 (20.8)	24 (24,24)	1.09 (2.33)	1.22 (4.38)
120	30	924 (21.6)	55400 (20.8)	24 (24,24)	30	1010 (21.1)	68900 (22.3)	24 (24,24)	1.1 (2.48)	1.24 (4.73)
140	30	1110 (22)	68300 (21.7)	24 (24,24)	30	1220 (21.6)	86500 (23.4)	24 (24,24)	1.11 (2.67)	1.26 (5.02)

N:number of observations; C_max_: maximum plasma concentration; AUC_0-168h_: area under the concentration time-curves from time 0 to 168 hours; T_max_: time to reach maximum concentration; RC_max_: the ratio of C_max_ after the first dosing to C_max_ after the last dosing; RAUC_0-168h_: ratio of AUC_0-168h_ in the first dosing to AUC_0-168h_ in the last dosing. T_max_ was shown as medium (minimum, maximum). Other values were shown as mean (standard deviation/mean*100).

The concentration-time curve within one dosing interval showed slightly elevated YPEG-rhGH concentration after the last dosing compared with that after the first dosing (**Figure 1C**). After the first dosing, the maximum plasma concentration (C_max_) of YPEG-rhGH was 766, 924, and 1110 ng/ml for YPEG-rhGH 100, 120 and 140 μg/kg/week, respectively; C_max_ of YPEG-rhGH was 811, 1010 and 1220 ng/ml after the last dosing, respectively, for the YPEG-rhGH groups (**Table 1**). A dose-dependent response of C_max_ and area under the concentration-time curves from time 0 to 168 hours (AUC_0-168h_) were seen for YPEG-rhGH. Moreover, the ratio of C_max_ after the first dosing to C_max_ after the last dosing was 1.09~1.11, and the ratio of AUC_0-168h_ in the first dosing to AUC_0-168h_ in the last dosing was 1.22~1.26. These two accumulation indices, consistent with the comparable PK profiles of YPEG-rhGH between the first and last dosing in all 3 groups (**Figure 1C**), showed no obvious accumulation of YPEG–rhGH after multiple dosing.

#### Model-derived pharmacokinetic/pharmacodynamic profile of IGF-1

**Figure 2:**
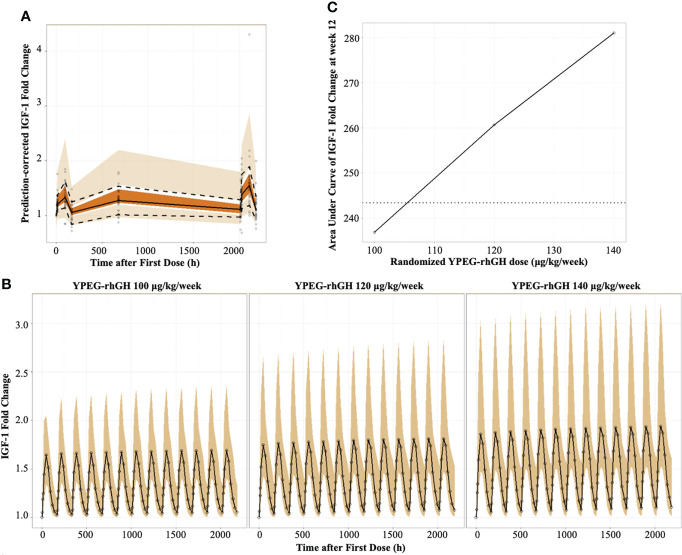
Model-derived PD profiles of YPEG-rhGH **(A)**, Prediction-corrected visual predictive checks (pcVPC) of IGF-1 fold change (IGF-1 FC). The hollow dots indicated the prediction-corrected IGF-1 FC. The top dashed line, the middle solid line and the bottom dashed line indicated 10th percentile, median and 90th percentile of prediction-corrected IGF-1 FC. The top, middle and bottom area indicated the 95% confidence interval of each line. **(B)**, model-derived PK/PD profiles of IGF-1FC. The hollow dots and solid line indicated the medium of IGF-1 FC. The shallow area indicated the 5% and 95% confidence intervals predicted by the model. **(C)**, the model-derived AUC-dose relationship of IGF-1 FC in GHD children. The black solid line and hollow dots indicated the geometric means of AUC_IGF-1 FC_ with YPEG-rhGH. The dashed line indicated the geometric means of AUC_IGF-1 FC_ with daily rhGH.

The indirect pharmacodynamic response model was constructed based on IGF-1 FC. Prediction-corrected visual predictive checks (pcVPC) showed a good estimation of IGF-1 FC by the model (**Figure 2A**). The IGF-1 FC-time curve showed that the IGF-1 levels increased initially and declined with time within one dosing interval. Compared with the first dosing, the IGF FC increased slightly after multiple dosing and reached a steady state after the 12th dose (**Figure 2B**). This data suggested that there was no obvious accumulation of IGF-1 after multiple dosing.

As described in **Table 2**, a dose-dependent relationship between YPEG-rhGH and the area under effect curve (AUEC) of IGF-1 FC was observed both at the first and last dosing, indicating that IGF-1 FC was a reliable indicator for YPEG-rhGH exposure. Moreover, the median T_max_ of IGF-1 FC was 48 h, while the median T_max_ of YPEG-rhGH was 24 hours (**Table 2**), further suggesting that the increase of IGF-1 levels lag behind the increase of YPEG-rhGH concentration.

**Table 2:** The model derived PK/PD parameters of IGF-1 FC.

Dose (μg/kg/week)	Week 1					Week 12					RC_max_	RAUEC_0-168h_
	N	C_max_ (ng/ml)	AUEC_0-168h_ (ng·h/ml)	T_max_ (h)	T_EC50_ (h)	N	C_max_ (ng/ml)	AUEC_0-168h_ (ng·h/ml)	T_max_ (h)	T_EC50_ (h)		
100	30	1.69 (17.6)	221 (9.76)	48 (24, 72)	18.5 (15, 24)	30	1.93 (54.1)	242 (24.8)	48 (24, 72)	19 (15,24)	1.11 (28.9)	1.09 (18.3)
120	30	2.14 (81.7)	254 (44.8)	48 (48, 72)	19 (16, 24)	30	2.4 (111)	280 (59.7)	48 (48, 72)	19 (16, 24)	1.06 (7.41)	1.08 (5.52)
140	30	2.5 (118)	280 (64.8)	48 (48, 72)	19 (16, 24)	30	2.77 (140)	311 (76.7)	48 (48, 72)	19.5 (16, 24)	1.06 (4.64)	1.09 (4.1)

N:number of observations; C_max_: maximum plasma concentration; AUEC_0-168h_: area under the effective curves from time 0 to 168 hours; T_max_: time to reach maximum concentration; T_EC50_: time to reach 50% of C_max_; RC_max_: the ratio of C_max_ after the first dosing to C_max_ after the last dosing; RAUEC_0-168h_: ratio of AUEC_0-168h_ in the first dosing to AUEC_0-168h_ in the last dosing. T_max_ and T_EC50_ were shown as medium (minimum, maximum). Other values were shown as mean (standard deviation/mean*100).

To confirm the corresponding dose of weekly YPEG-rhGH with daily rhGH, the geometric mean AUEC of IGF-1 FC (AUEC_IGF-1 FC_) was compared between weekly YPEG-rhGH with daily rhGH (**Figure 2C**). The data showed that the geometric mean AUEC_IGF-1 FC_ was comparable between YPEG-rhGH 106 μg/kg/week and daily rhGH, suggesting the corresponding dose of YPEG-rhGH with daily rhGH 35 μg/kg/day was 106 μg/kg/week. Considering the calculation bias due to the small fluctuation of daily rhGH, the efficacy of YPEG-rhGH 110 ~120 μg/kg/week could be comparable with daily rhGH 35 μg/kg/day.

**Figure 3:**
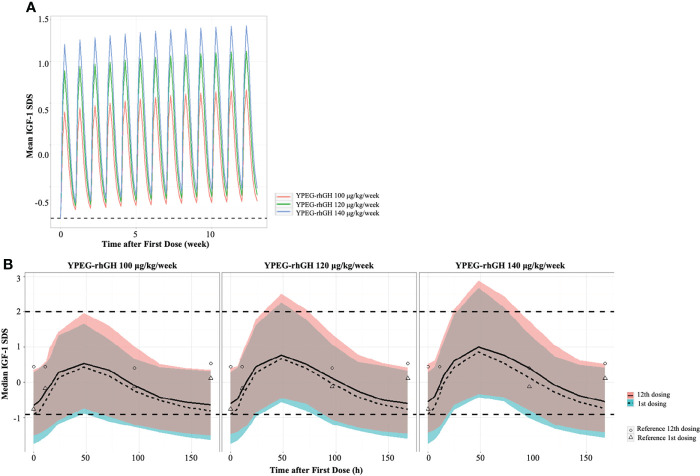
Model derived profile of IGF-1 SDS. **(A)**, the model-derived pattern of IGF-1 SDS during dose interval of YPEG-rhGH. **(B)**, model-derived PK/PD profiles of IGF-1 SDS. The green and pink area indicated the 95% confident interval of IGF-1 SDS after the first dose and last dose of YPEG-rhGH. The hollow triangles and dots indicated the mean IGF-1 SDS after the first and last dosing of daily rhGH respectively.

IGF-1 SDS is often used to monitor the safety of rhGH treatment. To explore the safety of YPEG-rhGH in respect with IGF-1 SDS, the average levels of IGF-1 were derived by the PK/PD model, and IGF-1 SDS was converted according to the age of the subject (20). As shown in **Figure 3A**, in 12 weeks of the treatment period, all the model-derived mean IGF-1 SDS values were between -2 and 2. This suggested good tolerance and safety of YPEG-rhGH treatment of 100, 120 and 140 μg/kg/week. The model-derived IGF-I SDS levels within one dosing interval were shown in **Figure 3B**. The time for IGF-I SDS value reached the peak was about 50 h after dosing, with a fluctuation greater than that of daily rhGH over a dosing interval (**Figure 3B**). However, at some time-points, the upper limits of 95% prediction interval of IGF-I SDS value in YPEG-rhGH 120 and 140 μg/kg/week were greater than 2, which suggested that safety monitoring might be needed for some patients treated with YPEG-rhGH 120 and 140 μg/kg/week.

### Efficacy

A total of 43 patients were randomly assigned to receive YPEG-rhGH or daily rhGH. One child in the daily rhGH group discontinued the treatment due to rash and recovered soon after developing symptoms. All of the 43 patients were included in the analysis. There was no significant difference in baseline characteristics between groups (**Table 3**). Children in the YPEG-rhGH 140 μg/kg/week group were relatively younger and shorter with lower IGF-1 levels, while HV, MPH (mid-parental height), GH peak levels were comparable between groups.

**Table 3:** Patients baseline characteristics in FAS.

	YPEG-rhGH100 μg/kg/week (A)	YPEG-rhGH120 μg/kg/week (B)	YPEG-rhGH140 μg/kg/week(C)	daily rhGH35 μg/kg/day(D)	Intergroup comparison (*P* value)		
					A vs D	B vs D	C vs D
n	10	12	9	12			
Sex, n (%)					1.000^[F]^	1.000^[F]^	1.000^[F]^
Male	7 (70.0%)	8 (66.7%)	6 (66.7%)	9 (75.0%)			
Female	3 (30.0%)	4 (33.3%)	3 (33.3%)	3 (25.0%)			
Age (year)	8.50 (7.00, 10.00)	7.00 (5.50, 8.50)	6.00 (4.50, 8.00)	7.00 (5.00, 8.00)	0.053^[T]^	0.488^[T]^	0.589^[T]^
Height (cm)	119.60 (109.60, 126.20)	112.55 (103.35, 122.85)	98.30 (94.90, 115.00)	113.20 (98.30, 121.70)	0.191^[T]^	0.460^[T]^	0.385^[T]^
Weight(kg)	20.3(4.6)	19.7(4.9)	17.0(4.3)	18.1(3.7)	0.2312^[A]^	0.3689^[A]^	0.5615^[A]^
BMI (kg/m^2^)	14.9(1.1)	15.2(1.3)	15.1(1.5)	14.8(1.0)	0.7983^[A]^	0.3469^[A]^	0.5348^[A]^
MPH (cm)	162.130 (3.3648)	161.004 (5.1470)	161.306 (3.6841)	162.979 (4.0253)	0.637^[A]^	0.254^[A]^	0.369^[A]^
HV (cm/year)	3.77 (1.989)	3.33 (0.958)	3.94 (1.634)	3.57 (1.371)	0.753^[A]^	0.695^[A]^	0.571^[A]^
HTSDS	-2.912(0.8576)	-2.558(0.5225)	-2.998(0.9323)	-2.353(0.5183)	0.220^[A]^	0.937^[A]^	0.146^[A]^
Bone age (year)	6.0 (4.5, 8.0)	5.0 (3.0, 6.0)	3.5 (2.0, 5.0)	4.0 (3.0, 5.5)	0.076^[T]^	0.527^[T]^	0.526^[T]^
GH peak, n (%)					1.000^[F]^	1.000^[F]^	0.659^[F]^
≤ 5 µg/L	3 (30.0%)	3 (25.0%)	2 (22.2%)	4 (33.3%)			
5 ~10 µg/L	7 (70.0%)	9 (75.0%)	7 (77.8%)	8 (66.7%)			
IGF-1 (ng/ml)	137.33 (52.133)	112.08 (40.210)	83.22 (41.745)	112.97 (41.416)	0.202^[A]^	0.961^[A]^	0.132^[A]^

Normally distributed data are shown as mean (SD) unless otherwise stated. Non-normal distributed data are shown as medium (first quartile, third quartile). ^[F]^Fisher’s exact test. ^[T]^Wilcoxon Rank Sum Test. ^[A]^one-way analysis of variance (ANOVA).FAS, Full analysis set; HTSDS, height standard deviation score; BMI, body mass index; MPH, mid-parental height; HV, height velocity; GH, growth hormone; IGF-1, insulin-like growth factor-1.

**Figure 4:**
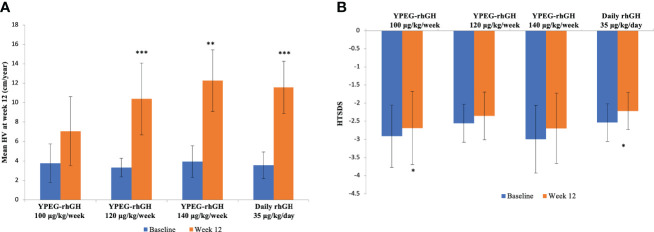
HV and HTSDS before and after the treatments. **(A)**, Mean HV at baseline and Week 12. **(B)**, HTSDS at baseline and Week 12. Data are shown as mean ± SD. *p <0.05, ** *p <*0.01, *** *p <*0.001.

After 12 weeks of treatment, there were no changes in body weight compared with the baseline (**Figure S1**). At week 12, the estimated annualized HVs for YPEG-rhGH 100, 120 and 140 μg/kg/week and daily rhGH was 7.07 ± 3.55 cm, 10.39 ± 3.70 cm, 12.27 ± 3.17, and 11.58 ± 2.68 cm/year respectively. Compared to baseline, YPEG-rhGH 100 μg/kg/week group showed an increasing trend of HV (*p* = 0.064), while the other 3 groups showed significant increases in HV (*p* = 0.004 in YPEG-rhGH 140 μg/kg/week group, *p <* 0.001 in YPEG-rhGH 120 μg/kg/week group and the daily rhGH group) (**Figure 4A**). Also, HV improvement was comparable between the treatments of YPEG-rhGH 120, 140 μg/kg/week and daily rhGH, but significantly lower in YPEG-rhGH 100 μg/kg/week group (*p* = 0.005) (**Table 4**).

**Table 4:** Statistical analysis of improvement of HV and HTSDS after 12 weeks of treatment.

	Group A (YPEG-rhGH100 μg/kg/week)	Group B (YPEG-rhGH120 μg/kg/week)	Group C (YPEG-rhGH140 μg/kg/week)	Group D (daily rhGH35 μg/kg/day)	Intergroup comparison(*P* value)		
					A vs D	B vs D	C vs D
Change in HV from baseline (cm/year)	3.30 (4.576)	7.07 (4.054)	8.32 (4.478)	6.22 (4.702)	0.005^[A]^	0.297^[A]^	0.494^[A]^
Change in HTSDS from baseline	0.228 (0.2596)	0.210 (0.3385)	0.304 (0.4388)	0.283 (0.3783)	0.754^[A]^	0.631^[A]^	0.867^[A]^

Data are shown as mean (SD). ^[A]^one-way analysis of variance (ANOVA).HV, height velocity; HTSDS, height standard deviation score.

Compared with the baseline, HTSDS at week 12 was significantly higher in YPEG-rhGH 100 μg/kg/week (-2.912 ± 0.858 before treatment, -2.685 ± 1.010 after treatment, *p* = 0.037) and daily rhGH (-2.535 ± 0.518 before treatment, -2.216 ± 0.511 after treatment, *p* = 0.042) groups (**Figure 4B**). There was a trend of increased HTSDS in YPEG-rhGH 120 μg/kg/week (-2.558 ± 0.523 before treatment, -2.348 ± 0.657 after treatment, *p* = 0.077) and 140 μg/kg/week (-2.998 ± 0.932 before treatment, -2.694 ± 0.966 after treatment *p* = 0.077 and 0.098 respectively) groups. The change in HTSDS before and after the treatment was comparable between groups (**Table 4**).

Treatment adherence was slightly higher for all groups of YPEG-rhGH treatment (**Table 5**). The adherence was relatively low in the daily rhGH group, due to one child who discontinued the treatment after 11 injections due to rashes (**Table 5**).

**Table 5:** Treatment adherence over 12 weeks of treatment in FAS.

	Group A (YPEG-rhGH100 μg/kg/week)	Group B (YPEG-rhGH120 μg/kg/week)	Group C (YPEG-rhGH140 μg/kg/week)	Group D (daily rhGH35 μg/kg/day)
MEAN (SD)	99.167 (2.6352)	100.000 (0.0000)	100.000 (0.0000)	92.063 (25.2810)
MEDIAN	100.000	100.000	100.000	100.000
RANGE	91.67, 100.00	100.00, 100.00	100.00, 100.00	11.90, 100.00

### Safety

The proportion of patients who reported adverse events (AEs) during the 12-week treatment period was 77.4% (24/31) and 75.0% (9/12), in YPEG-rhGH and daily rhGH groups respectively (**Table 6**). Among YPEG-rhGH treated patients, the proportion was similar between 3 doses (**Table 6**). Adverse events were mainly mild to moderate (Grade 1 and Grade 2). Related AEs were similar between YPEG-rhGH (29.0%, 9/31) and the daily rhGH group (33.3%, 4/12) (**Table 6**), including injection-site reaction, rashes, palpebral edema, etc. (**Table 7**). No unexpected adverse reactions occurred.

**Table 6:** Adverse events in safety analysis set.

	YPEG-rhGH								Daily rhGH	
	100 μg/kg/week(n=10)		120 μg/kg/week(n=12)		140 μg/kg/week(n=9)		Merged(n=31)		35 μg/kg/day(n=12)	
	P (%)	E	P (%)	E	P (%)	E	P (%)	E	P (%)	E
**All AEs**	7 (70.0)	17	10 (83.3)	23	7 (77.8)	32	24 (77.4)	72	9 (75.0)	27
Grade 1	3 (30.0)	11	8 (66.7)	15	6 (66.7)	18	17 (54.8)	44	5 (41.7)	9
Grade 2	4 (40.0)	6	4 (33.3)	8	5 (55.6)	13	13 (41.9)	27	6 (50.0)	18
Grade 3	0 (0.0)	0	0 (0.0)	0	1 (11.1)	1	1 (3.2)	1	0 (0.0)	0
Grade 4	0 (0.0)	0	0 (0.0)	0	0 (0.0)	0	0 (0.0)	0	0 (0.0)	0
Grade 5	0 (0.0)	0	0 (0.0)	0	0 (0.0)	0	0 (0.0)	0	0 (0.0)	0
**Related AEs**	2 (20.0)	10	4 (33.3)	6	3 (33.3)	3	9 (29.0)	19	4 (33.3)	4
Grade 1	2 (20.0)	10	2 (16.7)	4	3 (33.3)	3	7 (22.6)	17	2 (16.7)	2
Grade 2	0 (0.0)	0	2 (16.7)	2	0 (0.0)	0	2 (6.5)	2	2 (16.7)	2
Grade 3	0 (0.0)	0	0 (0.0)	0	0 (0.0)	0	0 (0.0)	0	0 (0.0)	0
Grade 4	0 (0.0)	0	0 (0.0)	0	0 (0.0)	0	0 (0.0)	0	0 (0.0)	0
Grade 5	0 (0.0)	0	0 (0.0)	0	0 (0.0)	0	0 (0.0)	0	0 (0.0)	0
**All SAEs**	0 (0.0)	0	0 (0.0)	0	1 (11.1)	1	1 (3.2)	1	0 (0.0)	0
**Related SAEs**	0 (0.0)	0	0 (0.0)	0	0 (0.0)	0	0 (0.0)	0	0 (0.0)	0
**AE related with discontinuation**	0 (0.0)	0	0 (0.0)	0	0 (0.0)	0	0 (0.0)	0	1 (8.3)	1

%, proportion of patients; E, number of events; AE, adverse event; SAE, serious adverse event; P, Number of patients;

**Table 7:** Related adverse events in safety analysis set.

MedDRA system organ classes	YPEG-rhGH								Daily rhGH	
	100 μg/kg/week(n=10)		120 μg/kg/week(n=12)		140 μg/kg/week(n=9)		Merged(n=31)		35 μg/kg/day(n=12)	
	P (%)	E	P (%)	E	P (%)	E	P (%)	E	P (%)	E
**General disorders and administration site conditions**	2 (20.0)	10	1 (8.3)	2	0 (0.0)	0	3 (9.7)	12	0 (0.0)	0
Injection-site pain	1 (10.0)	3	1 (8.3)	1	0 (0.0)	0	2 (6.5)	4	0 (0.0)	0
Injection-site edema	0 (0.0)	0	1 (8.3)	1	0 (0.0)	0	1 (3.2)	1	0 (0.0)	0
Injection-site mass	1 (10.0)	2	0 (0.0)	0	0 (0.0)	0	1 (3.2)	2	0 (0.0)	0
Injection-site swelling	1 (10.0)	3	0 (0.0)	0	0 (0.0)	0	1 (3.2)	3	0 (0.0)	0
Chest pain	1 (10.0)	2	0 (0.0)	0	0 (0.0)	0	1 (3.2)	2	0 (0.0)	0
**Skin and subcutaneous tissue disorders**	0 (0.0)	0	1 (8.3)	1	0 (0.0)	0	1 (3.2)	1	1 (8.3)	1
rash	0 (0.0)	0	1 (8.3)	1	0 (0.0)	0	1 (3.2)	1	1 (8.3)	1
**Eye disorders**	0 (0.0)	0	0 (0.0)	0	1 (11.1)	1	1 (3.2)	1	1 (8.3)	1
palpebral edema	0 (0.0)	0	0 (0.0)	0	1 (11.1)	1	1 (3.2)	1	1 (8.3)	1
**Immune system disorders**	0 (0.0)	0	1 (8.3)	1	0 (0.0)	0	1 (3.2)	1	0 (0.0)	0
hypersensitivity	0 (0.0)	0	1 (8.3)	1	0 (0.0)	0	1 (3.2)	1	0 (0.0)	0
**Endocrine disorders**	0 (0.0)	0	0 (0.0)	0	0 (0.0)	0	0 (0.0)	0	1 (8.3)	1
hypothyroidism	0 (0.0)	0	0 (0.0)	0	0 (0.0)	0	0 (0.0)	0	1 (8.3)	1
**Musculoskeletal and connective tissue disorders**	0 (0.0)	0	0 (0.0)	0	1 (11.1)	1	1 (3.2)	1	0 (0.0)	0
arthritis	0 (0.0)	0	0 (0.0)	0	1 (11.1)	1	1 (3.2)	1	0 (0.0)	0
**Investigations**	0 (0.0)	0	1 (8.3)	1	0 (0.0)	0	1 (3.2)	1	0 (0.0)	0
Low adrenocorticotropic hormone level	0 (0.0)	0	1 (8.3)	1	0 (0.0)	0	1 (3.2)	1	0 (0.0)	0
**Nervous system disorders**	0 (0.0)	0	0 (0.0)	0	1 (11.1)	1	1 (3.2)	1	0 (0.0)	0
idiopathic intracranial hypertension	0 (0.0)	0	0 (0.0)	0	1 (11.1)	1	1 (3.2)	1	0 (0.0)	0
**Cardiac disorders**	0 (0.0)	0	1 (8.3)	1	0 (0.0)	0	1 (3.2)	1	0 (0.0)	0
angina	0 (0.0)	0	1 (8.3)	1	0 (0.0)	0	1 (3.2)	1	0 (0.0)	0
**Gastrointestinal disorders**	0 (0.0)	0	0 (0.0)	0	0 (0.0)	0	0 (0.0)	0	1 (8.3)	1
Labial edema	0 (0.0)	0	0 (0.0)	0	0 (0.0)	0	0 (0.0)	0	1 (8.3)	1

%, proportion of patients; E, number of events; P, number of patients;

During the treatment, 1 patient in the daily rhGH group discontinued the drug after 11 days of treatment, due to rash that was probably related to the treatment. One serious adverse event (SAE) was reported in 1 child from the YPEG-rhGH 140 μg/kg/week group, because of hospitalization for acute bronchitis which was probably not related to the treatment.

The rates of injection-site reactions in YPEG-rhGH were higher compared with daily rhGH. There were 2 children in the YPEG-rhGH groups and no cases in the daily rhGH group who reported related injection-site reactions (**Table 7**). These patients recovered soon after the treatment. Additionally, lipoatrophy, which was reported in a pilot study of the once-weekly PEGylated GH formulation PHA-794428 (21), was not reported during the treatments of YPEG-rhGH.

The treatments did not affect glucose and lipid metabolism in any of the groups. No hyper/hypothyroidism was reported (**Table S1**).

## Discussion

PEGylation is a versatile drug delivery technique. Recently, a novel Y-shape branched PEGylated rhGH (YPEG-rhGH) was formulated, providing higher protection of rhGH and relatively higher bioactivity of the protein. The previous phase 1 trial showed that the mean half-life of YPEG-rhGH was 65-120 hours (data not shown). The PK/PD model derived from this study demonstrated that there was no obvious accumulation of YPEG-rhGH and IGF-1 SDS was in the normal range. After 12-week treatment, the estimated annualized HV in YPEG-rhGH 120, and 140 μg/kg/week groups were comparable with that of daily rhGH, with similar safety and tolerability. These data indicated that YPEG-rhGH could be suitable for once-weekly treatment in children with GHD, with 120 ~ 140 μg/kg/week as the optimal dose.

The population PK/PD model derived from this study showed the YPEG-rhGH concentration increased slightly after multiple dosing and reached a steady state after the 12th dose. C_max_ after the first dosing to C_max_ after the last dosing was 1.09~1.11, and the ratio of AUC_0-168h_ in the first dosing to AUC_0-168h_ in the last dosing was 1.22~1.26 in YPEG-rhGH groups, suggesting no obvious accumulation of YPEG–rhGH after multiple dosing. Regarding the PD profile, IGF-1 FC was chosen as the parameter to adjust the differences in basal IGF-1 levels. We found that IGF FC slightly increased after multiple dosing and reached a steady state after the 12th dose. The PD/PK modeling showed a dose-dependent relationship between IGF-1 and YPEG-rhGH. Regarding the estimated change of IGF-1 levels, the efficacy of YPEG-rhGH 110~120 μg/kg/week was similar to that of daily rhGH 35 μg/kg/day. These data indicated that YPEG-rhGH possesses a suitable profile for once-weekly treatment.

One major concern about LAGH is that the constantly elevated GH levels might imply a risk for supraphysiological IGF-I levels, and consequently increase the risk of neoplasia, acromegaly, and glucose intolerance (9, 22). The PK/PD model showed that IGF-I SDS increased to a peak value at about 50 h after dosing, with a fluctuation greater than that of daily rhGH. In the YPEG-rhGH group, the model-derived average IGF-1 SDS peak values were above and trough values were below the average IGF-I levels in daily rhGH group. The PK/PD model also indicated that the peak IGF-1 SDS values in YPEG-rhGH 120 and 140 μg/kg/week might be above 2 for a transient period (days 2-3), suggesting patients treated with these two doses should be closely monitored.

Inferior efficacy is one of the theoretical suspicions of LAGH. The physiologic secretory pattern of GH occurs in an episodic and pulsatile pattern. Elevated and non-pulsatile GH exposure in LAGH treatment may downregulate or desensitize GH receptor signaling and result in inferior efficacy of LAGH (23, 24). In this study, the efficacy of YPEG-rhGH treatment was dose-dependent. HV was significantly increased after 12 weeks of treatment of YPEG-rhGH 120 and 140 μg/kg/week. The increase of HV in YPEG-rhGH 120 μg/kg/week and 140 μg/kg/week groups was comparable to that of the daily rhGH group. The change in HV SDS was comparable between YPEG-rhGH 100, 120 and 140 μg/kg/week and daily rhGH. Thus, we concluded that YPEG-rhGH demonstrated non-inferior efficacy to daily rhGH in GHD patients. Based on HV, change in HV SDS, and model-derived IGF-I profiles, YPEG-rhGH dose for the phase 3 trial in children with GHD should be 120 ~ 140 μg/kg/week.

The spectrum and incidence of AEs were overall similar in patients treated with YPEG-rhGH and daily rhGH. YPEG-rhGH was well tolerated at all 3 doses investigated. Except for one unlikely related SAE (acute bronchitis), other AEs were mild to moderate. Although our study showed a slightly higher incidence of injection-site reactions with YPEG-rhGH, these AEs were mild and resolved soon after injection without any treatment, which suggested that the tolerability of YPEG-rhGH was consistent with that of daily rhGH. Additionally, adherence was slightly higher in the YPEG groups. These data suggested the similar safety and tolerability of YPEG-rhGH with daily rhGH, with no new significant safety or local tolerability issues.

Moreover, our previous patent showed YPEG-rhGH had higher bioactivity compared with N-terminal PEGylation, thus suggesting that the dose of YPEG-rhGH to achieve consistent efficacy with daily rhGH could be lower than Jintrolong^®^. This hypothesis was supported by the observation that the non-inferior dose of Jintrolong^®^ to daily rhGH 35 μg/kg/day was 200 μg/kg/week (11), and that of YPEG-rhGH was only 120 ~ 140 μg/kg/week. More data from the phase 3 trial are still be needed to further confirm the hypothesis.

This study has a few limitations. The samples size was relatively small and the treatment period was short. Phase 3 and the second period of phase 2 investigating the efficacy and safety of YPEG-rhGH are currently ongoing. Another limitation is that rhGH and PEG-rhGH antibody evaluations were not included in the current study. Several reports showed that anti-PEG antibodies accelerated the clearance of PEG-conjugated proteins and contribute to the loss of efficacy of PEG conjugates (25, 26). Therefore, we have included the measurement of these antibodies in the ongoing trials.

## Conclusions

The present data provide strong evidence that YPEG-rhGH is suitable for once-weekly treatment for children with GHD; 120 ~140 μg/kg/week of YPEG-rhGH is highly efficacious with a favorable safety profile. A phase 3 trial has been initiated to further investigate the efficacy and safety of once-weekly YPEG-rhGH in a large cohort of children with GHD.

## Data availability statement

The original contributions presented in the study are included in the article/**Supplementary Material**. Further inquiries can be directed to the corresponding authors.

## Ethics statement

The studies involving human participants were reviewed and approved by the Ethics Committee of Tongji Hospital, Tongji Medical College, Huazhong University of Science and Technology. Written informed consent to participate in this study was provided by the participants’ legal guardian/next of kin.

## Author contributions

Conceptualization: XL and LS. Methodology: HeZ, WZ, and RH. Data analysis: HeZ, WZ, and RH. Data collection: CZ, YL, HuW, HD, GZ, YY, HZ, HG, PL, FS, and ZX. Writing—original draft preparation: CZ and YL. Writing—review and editing: CZ, YL, LS, and XL. Supervision: XL and LS. All authors contributed to the article and approved the submitted version.

## Conflict of interest

LS is the president of Xiamen Amoytop Biotech Co., Ltd. RH and WZ are employees of Xiamen Amoytop Biotech Co., Ltd.

The remaining authors declare that the research was conducted in the absence of any commercial or financial relationships that could be construed as a potential conflict of interest.

## Publisher’s note

All claims expressed in this article are solely those of the authors and do not necessarily represent those of their affiliated organizations, or those of the publisher, the editors and the reviewers. Any product that may be evaluated in this article, or claim that may be made by its manufacturer, is not guaranteed or endorsed by the publisher.

## Supplementary material

Supplementary Figure 1 Body weight before and after the treatments. Data are shown as mean ± SD.
