# Supplementary figures and images for: The pharmacokinetic and pharmacodynamic properties and short-term outcome of a novel once-weekly PEGylated recombinant human growth hormone for children with growth hormone deficiency
Source: Front Endocrinol (Lausanne). 2022 Aug 11;13:922304. doi: 10.3389/fendo.2022.922304 (PMC9405430; doi:10.3389/fendo.2022.922304)

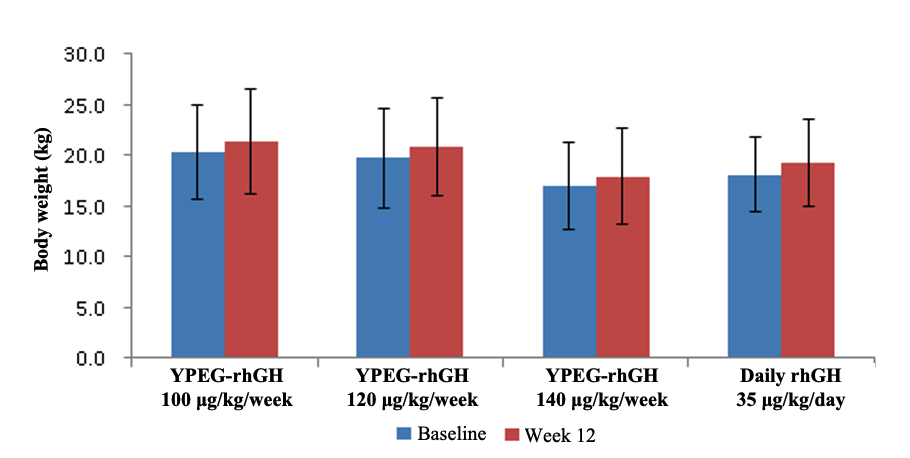

Supplement: Supplementary Figure 1 — Body weight before and after the treatments. Data are shown as mean ± SD. [file Image_1.tiff]
