# Supplementary material for: The pharmacokinetic and pharmacodynamic properties and short-term outcome of a novel once-weekly PEGylated recombinant human growth hormone for children with growth hormone deficiency
Source: Front Endocrinol (Lausanne). 2022 Aug 11;13:922304. doi: 10.3389/fendo.2022.922304 (PMC9405430; doi:10.3389/fendo.2022.922304)
Supplement: Supplementary file 2 [file Table_1.pdf]

Supplementary Table 1. Metabolic profile in safety analysis set.

	Group A (YPEG-rhGH 100 μg/kg/week)		Group B (YPEG-rhGH 120 μg/kg/week)		Group C (YPEG-rhGH 140 μg/kg/week)		Group D (daily rhGH 35 μg/kg/day)	
	Baseline	Week 12	Baseline	Week 12	Baseline	Week 12	Baseline	Week 12
Fasting blood glucose (mmol/L)	4.847	4.811	4.601	4.613	4.530	4.436	4.595	4.947
Fasting insulin (mIU/L)	5.369	6.613	4.511	4.120	3.514	3.343	3.755	6.921
Total cholesterol (mmol/L)	4.039	4.053	4.213	4.473	4.104	3.998	4.119	3.866
Triglyceride (mmol/L)	0.620	0.727	0.698	0.655	0.713	0.789	0.708	1.064
T3 (nmol/L)	2.367	2.499	2.175	2.248	2.328	2.582	2.382	2.637
T4 (mIU/L)	117.685	126.515	103.523	119.354	117.728	131.466	109.256	108.084
TSH (nmol/L)	2.642	3.421	2.693	2.991	2.684	3.412	2.313	2.557

Abbreviations: T3, triiodothyronine; T4, thyroxine; TSH, thyroid stimulating hormone.
